# Correction of paralytic hand deformities

**DOI:** 10.1186/1753-6561-9-S3-A72

**Published:** 2015-05-19

**Authors:** Sajid Husain

**Affiliations:** 1 Department of Plastic Surgery, JALMA Institute for Leprosy, Agra, 282006, India

## 

The ulnar nerve is most commonly involved in leprosy and is affected at or just above the elbow. The outcome of this damage is paralysis of the intrinsic muscles of the hand with the exception of the thenar group, together with paresis of ring and little finger flexor digitorum profundus (FDP). Although the obvious deformity is clawing of the fingers, other deformities are produced:

1. Hollowing of the intermetacarpal and thumb web space.

2. Flattening of the ulnar half of the hand.

3. Loss of abduction and adduction of the fingers.

4. Instability of metacarpophalangeal joint(MCPJ)

5. Flattening of the distal transverse metacarpal arch.

The overall effect is poor grip due unstable MCPJ, poor grasp because of inadequate opening of the hand, rolling of finger tips and a poor pinch due unstable MCPJ of the index finger.

Loss of function and anatomical defects needs surgical correction .The appearance of the hand is also very important for leprosy patients, because it is the characteristic deformity which is the principal cause of social rejection.

For every single deformity, there is an operative answer. But performing such a large number of operations defeats the very purpose for which these are performed. The goal should be to achieve the best for functional and social needs of the patients with the fewest interventions.

The choice for surgery involves a consideration of disease status, condition of the hand, age, extent of paralysis, occupational needs and also the socio-economic background.

## These patients can be operated provided they

1. Have taken anti leprosy treatment at least for six months.

2. Free from lepra reactions.

3. Hand should be fully mobile and free from contractures.

## What can be done

All four fingers require correction because the interossei are paralysed and these fingers need to be supported at the MCPJ.

The aim is to restore a balance between the flexor and extensor forces around the MCPJ. A number of procedures are available for correction of finger clawing. I am going to discuss the procedures which are most commonly used at our centre.

## Correction of clawing of fingers-

The available Motor unit for transfer are Palmaris Longus, Superficialis, Extensor carpi radials longus / Brevis. We prefer Palmaris longus if availabe or ECRL .The advantage of Palmaris longus (PL) is minimum morbidity to motor units. ECRL is a strong muscle and useful for stiff claws.

ECRL is withdrawn near the snuff box over the wrist joint and elongated with fascia lata graft and divided into four slips .This is transferred to the palmer aspect of the hand and sutured to lateral bands to stabilise the MP joints of all four fingers. This ECRL transfer is very useful in patients who have a tendency to flex the wrist. The correction of Z deformity in the thumb can be taken care of by transfer of ½ Flexor pollicis longus (FPL) to Extensor pollicis longus (EPL)

Another problem in claw hand is depression of the 1st web space. In ulnar nerve palsy, the muscles adductor pollicis and 1st dorsal interosseous becomes atrophied. Since these muscles give fullness to the 1st web space, the atrophy of these muscles results in hollowing of the 1st web space producing visible deformity. Correction of finger clawing makes the deformity more obvious when the patients opens up his hand fully or shakes hand with someone. This makes the patients more conscious of his web space deformity.

Surgeons have tried silicon gel injections, autologous fat graft, dermofat graft to fill this depression with varying degree of success. We have planned an adipocutaneous flap based on the cephalic vein or its major tributary from the radial side of the lower forearm for transfer into the depression of the 1st web space.

